# Effects of a vegetable-eel-earthworm integrated planting and breeding system on bacterial community structure in vegetable fields

**DOI:** 10.1038/s41598-018-27923-y

**Published:** 2018-06-22

**Authors:** Xianqing Zheng, Weiguang Lv, Ke Song, Shuangxi Li, Hanlin Zhang, Naling Bai, Juanqin Zhang

**Affiliations:** 1 Institute of Eco-Environment and Plant Protection, Shanghai Academy of Agricultural Sciences, Shanghai, 201403 China; 2 Shanghai Scientific Observation and Experimental Station for Agricultural Environment and Land Conservation, Ministry of Agriculture of China, Shanghai, 201403 China; 3 Shanghai Key Laboratory of Protected Horticultural Technology, Shanghai, 201403 China

## Abstract

Agricultural production combined with planting and breeding, which can reduce chemical fertilizer and pesticide applications, reduce losses due to natural disasters, and improve the output and quality of agricultural products, is an important way to achieve green, circular and efficient production. To assess effects on soil bacterial community structure, a vegetable-eel-earthworm integrated planting and breeding platform (VEE-IPBP) combined with experiment planting was established at Chongming Island, Shanghai and compared to traditional planting. High-throughput sequencing to reveal soil bacterial community structure was performed on samples collected at 0, 3 and 6 years after implementation of the two models. Over time, the Shannon index first increased and then decreased in the VEE-IPBP system and was reduced by 3.2% compared to the traditional planting (In the same time and space scale, the single-degree planting method of dryland vegetables under mechanical cultivation is adopted) (*p* < *0.05*). In contrast, Chao and Ace indices were increased by 2.4% and 3.2%. Thus, soil bacterial diversity was markedly different in the two planting models. The abundance of *Proteus*, *Cyanophyta* and *Cyanophyta* in soil increased after 6 years, and the proportion of *Lysinibacillus* increased significantly, contributing to improvement in soil disease resistance. Redundancy analysis (RDA) showed that the soil pH and water content were the main factors influencing the change in soil bacterial community structure in the two planting models, and the dominant species of soil bacteria were *Lysobacter* and *Bacillus*.

## Introduction

Integrated planting and breeding is a circular eco-agricultural production approach that takes full advantage of ecological principles to achieve reasonable systematic allocation for matter–energy conversion. Indeed, integrated planting and breeding platforms (IPBPs) can help to increase agricultural resource utilization efficiency, protect agro-ecological environments and promote green agricultural development. IPBPs have been used extensively worldwide for some time, and there are a wide variety of IPBPs with varied functions. The economic, ecological and social benefits due to the implementation of IPBPs have recently been studied extensively^1–4^. In various countries and territories in Europe and the United States, grain production has been integrated with livestock and poultry breeding, e.g., forage-crop-cow, grain-vegetable-pig and rice-mushroom-goose IPBPs^5,6^. Research has shown that at a suitable scale, livestock and poultry breeding combined with grain and vegetation production is advantageous in that livestock and poultry excrement can be returned to the field after fermentation and composting. The effects on soil include increasing the nutrient content (e.g., organic matter (OM)), improving the physical structure and aeration, balancing the pH and significantly enhancing bacterial diversity. Furthermore, some countries in Asia have established systems by integrating aquaculture with traditional agricultural production (integrated agro-aquaculture), also with relatively high resource utilization efficiency and system productivity^7,8^. For example, rice-fish symbiotic systems have had the following impacts: increased rice production per unit area by 5–15%, organic fertilizer utilization efficiency, soil microbial populations and activities, and soil respiration; improved rice quality traits and soil physical and chemical properties; reduced chemical fertilizer and pesticide application, pests and diseases, and accumulation of harmful reducing substances. Overall, these changes alter the soil microbial community structure^9,10^.

An integrated model of vegetables, eels and earthworms (that is, VEE-IPBP) constitutes a farmland ecosystem comprising dryland vegetables, soil animals and benthic aquatic animals in a habitat in which water and drought coexist^11,12^. It is reported that a farmland ecosystem in which wet and dry fields coexist can increase yield per unit area by more than 50%, reduce economic losses from floods, pests and diseases by more than 30% and achieve 100% recycling and reuse of agricultural crop wastes (e.g., stalks)^13^. Most research to date on 3D VEE-IPBP has focused on reducing pesticide and chemical fertilizer application and greenhouse gas emissions and on increasing soil nutrient utilization efficiency, crop output, agricultural product quality, and use of agricultural crop wastes (e.g., stalks). However, studies on soil microbial diversity under VEE-IPBP have rarely been reported. Thus, the mechanism by which VEE-IPBP affects the soil microbial community structure remains unclear. Soil microbes are not only biotic agents responsible for soil formation but are also important components of the soil ecosystem and play a pivotal role in humus formation, OM decomposition and soil nutrient cycling and transformation^14,15^. With specific functions in soil matter transformation and energy flow, various physiological groups of soil microbes produce an active pool of plant nutrient elements^16,17^, and their presence and activity affect soil fertility and crop nutrient supply^18^. In fact, soil microbial diversity can reflect, with high sensitivity, the functional evolution of an ecosystem as well as environmental stress^19–21^, and it is the basis for sustainable farmland ecosystem development^22^. Therefore, by studying the species, communities and functional diversity of soil microbes in regional farmland, we can provide a basis for evaluating the rationality of planting systems in a certain area.

In this study, the multi-year effects of VEE-IPBP on soil microbial community structure and diversity were analysed using Illumina high-throughput sequencing technology, with the goal of providing basic parameters for improving VEE-IPBP management and control measures as well as a scientific basis for increasing soil fertility and reasonably and sustainably utilizing cultivated land resources.

## Results and Discussion

### Effects of IPBP on soil bacterial diversity indices

Mean Shannon index values of 6.08, 6.45, 6.44, 6.54 and 6.23 were obtained for soil samples TPP10, TPP13, TPP16, VEE13 and VEE16, respectively. These results indicate that diversity in the TPP-treated plots increased with time; in contrast, Shannon index values for VEE-IPBP-treated soils first increased and then decreased. Three years after implementation of the two planting systems, Shannon index values for soil in VEE-IPBP-treated plots (i.e., soil sample VEE13) were significantly higher than those in TPP-treated plots (i.e., soil sample TPP13). Although Shannon index values for VEE-IPBP-treated plots (i.e., soil sample VEE16) were significantly lower than those for soil in the TPP-treated plots (i.e., soil sample TPP16) (*p* < 0.05) after 6 years, that of the soil in both VEE-IPBP- and TPP-treated plots was higher than that of the basic soil (i.e., soil sample TPP10, collected before the beginning of the experiment). Species richness indices for the soil in plots under the two planting systems were also compared. Mean Chao index values for soil samples TPP10, TPP13, TPP16, VEE13 and VEE16 were 2,578, 3,027, 3,064, 3,133 and 3,138, respectively, and mean ACE index values for these samples were 2,583, 3,028, 3,062, 3,118 and 3,159, respectively (*p* < 0.05). Both indices increased as with time under the planting systems. In addition, soil bacterial species richness was higher in VEE-IPBP-treated plots than in TPP-treated plots (Table 1).

**Table 1:** Effects of VEE-IPBP on soil bacterial richness and diversity indices.

Treatment	Out	Ace	Chao	Shannon
TPP10	2019b	2583b	2578b	6.08d
TPP13	2431a	3028a	3027a	6.45b
VEE13	2495a	3118a	3133a	6.54a
TPP16	2505a	3062a	3064a	6.44b
VEE16	2514a	3159a	3138a	6.23c

Currently, opinions differ with regard to whether earthworms promote or inhibit bacterial biomass and diversity. Most studies have concluded that as a result of their activities and foraging, earthworms increase soil aeration, with the enzymes in their intestinal secretions creating an environment that is favourable for increasing the soil bacterial biomass and diversity^23–26^. However, a large number of studies have also found that after passing through the intestines of earthworms, no change or significant increase in bacterial biomass occurs. Moreover, due to their feeding habits, burrow compression or surface secretions, a decrease in bacterial diversity or activity may even occur for some species of earthworm^27–31^. The results obtained in this study showed that compared to TPP, an increase in soil bacterial richness but a decrease in soil bacterial diversity occurred in VEE-IPBP-treated plots. This result may be due to a certain amount of water remaining in the ditches in VEE-IPBP-treated plots throughout the year, resulting in a relatively high soil moisture content. This directly affects bacterial activity in the intestinal and body-surface secretions of *Pheretima guillemi* earthworms or indirectly influences bacterial diversity by affecting the form, ratio and content of soil nutrients. Another possible reason is that by burrowing in the soil or feeding on soil and aquatic animals, the introduction of swamp eels might have affected soil bacterial richness. Regardless, the effects of swamp eels on soil microbes require further validation due to the current lack of research on this subject.

**Figure 1:**
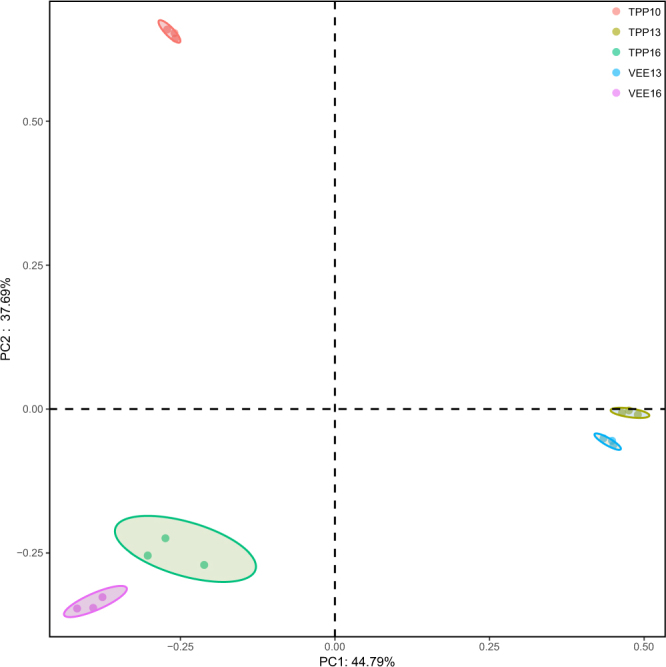
PCoA of the effects of different planting systems and durations on soil bacterial diversity.

UniFrac-based principal coordinate analysis (PCoA) can visually display similarities and differences in microbial evolution between different environmental samples^32^. Figure 1 shows the results of Bray-Curtis PCoA comparing changes occurring in the two planting systems from an evolutionary perspective. As indicated in Fig. 1, the different planting systems and durations can be satisfactorily distinguished, which also indicates that the presence and activities of earthworms and swamp eels significantly affected soil microbial diversity. However, determination of the specific mechanism involved requires further research.

### Effects of planting systems on soil bacterial community structure

**Figure 2:**
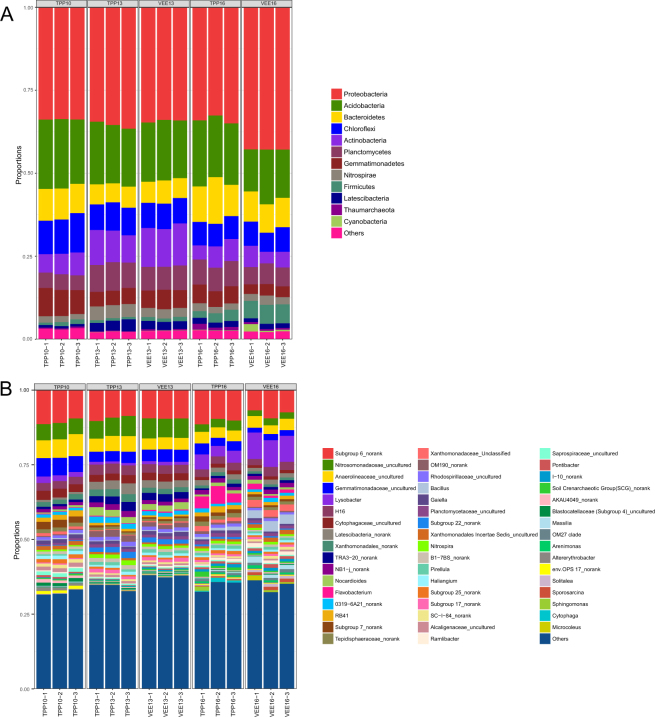
Analysis of soil bacterial community structure and composition in plots subjected to different planting systems (**A**) community structure and composition at the phylum level; (**B**) community structure and composition at the genus level).

The bacteria in the soil samples from the plots subjected to various treatments were grouped at the phylum level. Based on the abundance of sequences contained in different operational taxonomic units (OTUs) in soil samples from plots subjected to different treatments, a community structure histogram was produced after clustering (Fig. 2A), revealing differences in bacterial community structure and relative abundance at the phylum level. A total of 12 bacterial phyla with abundances greater than 1% were found in soil samples collected from TPP- and VEE-IPBP-treated plots, namely, Proteobacteria, Acidobacteria, Bacteroidetes, Chloroflexi, Actinobacteria, Planctomycetes, Gemmatimonadetes, Nitrospirae, Firmicutes, Latescibacteria, Thaumarchaeota and Cyanobacteria. Proteobacteria and Acidobacteria accounted for more than 60% of all phyla. An increase in the abundance of Proteobacteria, Firmicutes and Cyanobacteria was observed in VEE-IPBP-treated plots (i.e., soil sample VEE16) at 6 years after implementation of the planting systems. The majority of Proteobacteria and Firmicutes bacteria are facultative or obligate anaerobes and heterotrophs. Bacteria of the phylum Cyanobacteria are photosynthetic bacteria of the kingdom Eubacteria. The presence of ditches, which contained a certain amount of water throughout the year, in VEE-IPBP-treated plots may have created a suitable living environment for bacteria of the phylum Cyanobacteria, which showed increased abundance. Some researchers believe that although the environment in the digestive tracts of earthworms can help increase bacterial biomass, earthworms also feed on bacteria, resulting in a decrease in the population of certain bacterial phyla and an increase in anaerobic bacteria. This in turn results in a change in the composition of the bacterial communities in earthworm faeces, thereby affecting soil bacterial community composition^33^.

To further understand the functions of the bacteria present, the phyla in the soil samples collected from plots subjected to various treatments were clustered at the genus level. As indicated above, after clustering, a community structure histogram was produced based on the abundance of sequences contained in different OTUs (Fig. 2B). Genera with abundance ranked in the top five included *Subgroup 6_norank*, *Nitrosomonadaceae_uncultured*, *Anaerolineaceae_uncultured*, *Gemmatimonadaceae_uncultured* and *Lysobacter*. The abundance of *Lysobacter* in VEE-IPBP-treated plots had increased significantly at 6 years after implementation of the planting systems. Some researchers have found that *Lysobacter* bacteria can produce myxin^34^, and according to current research, most bacteria of this genus exert antagonistic effects toward pathogenic fungi, gram-positive (G^+^) bacteria, nematodes and green algae^35^. The sudden increase in the population of *Lysobacter* in the soil of VEE-IPBP-treated plots might be due to the “favourable” environment (suitable moisture content, neutral pH and high amino acid, low molecular weight sugar and fatty acid contents) of the earthworm intestines for growth^36^, possibly awakening dormant or inactive *Lysobacter* bacteria in the soil.

### Differences between soil bacterial community groups

**Figure 3:**
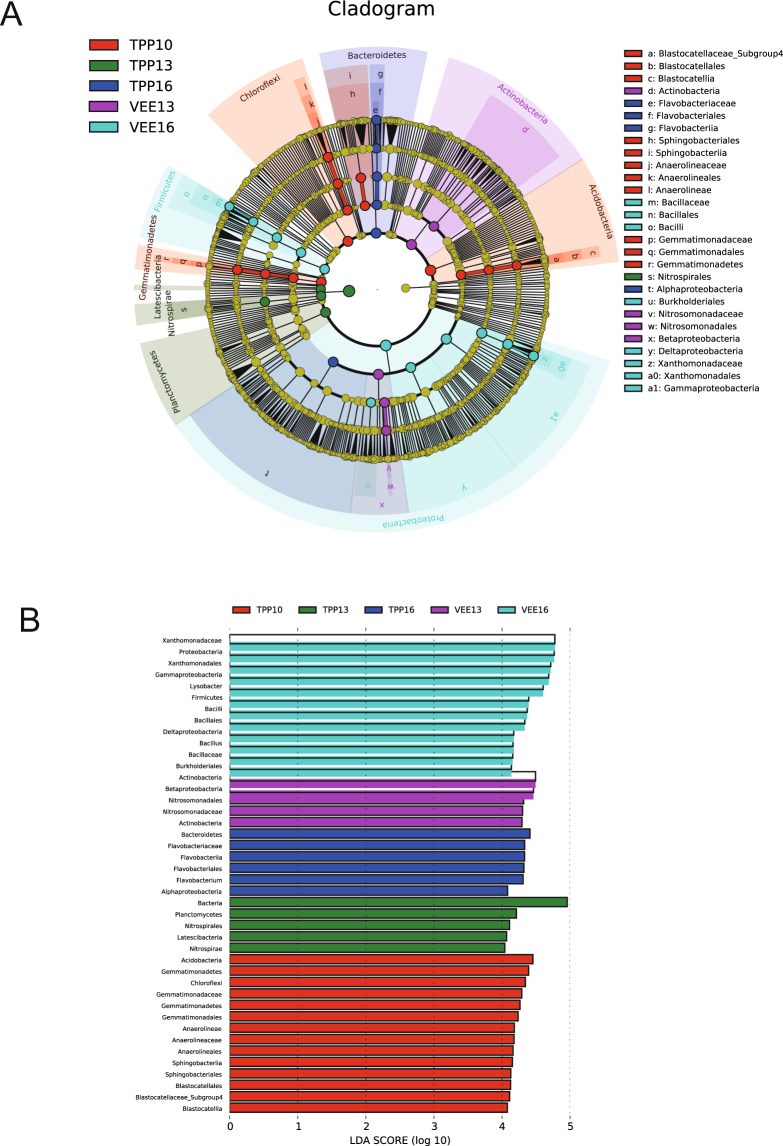
Analysis of differences in soil bacterial community composition between plots subjected to different planting systems (LDA score > 4).

LEfSe, an algorithm for high-dimensional biomarker discovery and explanation, can determine the genomic functions between biotic conditions using two, or more than two, different characteristics. We used linear discriminant analysis (LDA) to distinguish differences in bacterial groups between the two planting systems and years^37^. The results (Fig. 3A) showed 41 communities with an LDA score greater than 4 (Fig. 3B). The distribution of communities with phylogenetic differences in soil samples collected from plots subjected to five different treatments was as follows: 10 at the phylum level, 11 at the class level, 10 at the order level, 7 at the family level and 3 at the genus level. At the phylum level, 3 phylogenies of bacteria in soil sample TPP10 (i.e., basic soil sample) exhibited relatively high relative abundance, namely, Acidobacteria, Chloroflexi and Gemmatimonadetes. After 3 years, Planctomycetes, Nitrospirae and Latescibacteria showed relatively high relative abundance in TPP-treated plots (i.e., soil sample TPP13); after 6 years, Bacteroidetes became the phylogeny with relatively high relative abundance (i.e., soil sample TPP16). Many species of Bacteroidetes reside in the intestines of humans and animals; most are pathogenic and be shed in faeces. In this study, bacteria of the phylum Bacteroidetes in the soil might have originated from the organic fertilizer applied, remaining and gradually becoming the dominant bacteria^38^. However, soil in VEE-IPBP-treated plots was notable for the presence and activities of earthworms and swamp eels, which adjusted soil bacterial community composition indirectly by affecting physical indices of the soil (nutrient content, element ratio, volume weight, porosity and pH) or directly by feeding on bacteria, such as those of Bacteroidetes, or providing a suitable growth environment for Proteobacteria, Actinobacteria and Firmicutes. Liu *et al*.^39^ and Liu *et al*.^40^ have found that soil environments with a high moisture content are more suitable for bacteria of the phylum Proteobacteria. In the present study, we found a relatively high relative abundance of Actinobacteria in the soil of VEE-IPBP-treated plots after 3 years (i.e., soil sample VEE13), with Proteobacteria and Firmicutes showing high relative abundance after 6 years (i.e., soil sample VEE16).

### Redundant analysis of microbial communities and soil environmental factors

**Figure 4:**
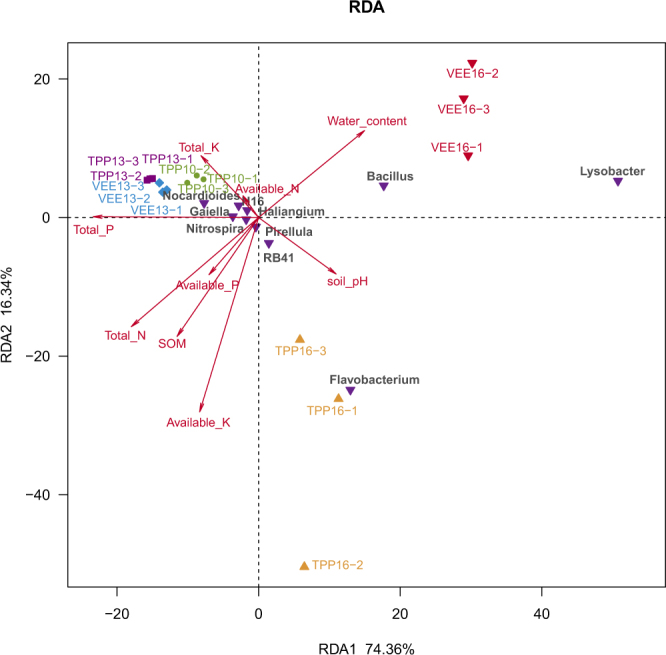
Analysis of correlations between soil bacterial communities in TPP- and VEE-IPBP-treated plots and physical and chemical properties of soil.

Correlations between soil bacterial community structure in VEE-IPBP- and TPP-treated plots and the physical and chemical properties of soil were analysed (Table 2), with explanatory power of 90.70% for all physical and chemical property variables for the different planting systems and durations (Fig. 4). Changes in the 10 main bacterial genera are most significantly correlated to the available potassium (AK) content of the soil, followed by the total phosphorous (TP), total nitrogen (TN), moisture and available N (AN) contents. Each planting systems caused changes in soil nutrients, thereby affecting soil bacterial communities. At the beginning of and at 3 years after implementation of VEE-IPBP, *Nocardioides*, *Haliangium*, *Gaiella* and *Nitrospira* were the main bacterial genera due to significant effects on TP, TK and TN contents. As the planting duration extended, each planting systems significantly affected soil physical and chemical properties as well as bacterial genera. Because of strong effects of soil pH, *Flavobacterium* became the main bacteria in soil of TPP-treated plots (i.e., soil sample TPP16) after 6 years. In comparison, the bacteria in VEE-IPBP-treated plots (i.e., soil sample VEE16) were mainly significantly affected by the soil moisture content after 6 years (*p* < 0.001), and *Lysobacter* and *Bacillus* became the main genera composing the soil bacterial community structure in these plots. These findings further demonstrate the effects of soil pH adjusted by earthworms and of the aquatic environment by aquaculture of swamp eels on soil bacterial communities in VEE-IPBP-treated plots.

**Table 2:** Physical and chemical properties of soils from different planting systems.

Parameter	Treatment				
	TPP10	TPP13	VEE13	TPP16	VEE16
SOM (g·kg^−1^)	26.63 ± 1.82a	23.03 ± 0.72b	19.43 ± 2.64c	25.80 ± 0.65a	19.10 ± 2.25c
Total N (g·kg^−1^)	2.17 ± 0.17a	1.60 ± 0.29b	1.73 ± 0.18b	2.00 ± 0.02a	1.20 ± 0.01c
Total P (g·kg^−1^)	1.93 ± 0.17a	1.50 ± 0.13c	1.70 ± 0.07b	1.40 ± 0.01c	1.10 ± 0.04d
Total K (g·kg^−1^)	1.30 ± 0.05d	1.82 ± 0.03a	1.79 ± 0.03a	1.45 ± 0.01c	1.60 ± 0.02b
Available N (mg·kg^−1^)	119.00 ± 13.65a	33.61 ± 2.08c	34.97 ± 2.19c	47.66 ± 0.25b	48.85 ± 2.16b
Available P (mg·kg^−1^)	38.76 ± 11.14a	19.21 ± 4.64c	24.06 ± 5.37bc	29.25 ± 1.00b	18.85 ± 0.23c
Available K (mg·kg^−1^)	76.67 ± 5.77b	73.33 ± 5.77b	76.67 ± 15.28b	140.00 ± 3.20a	30.00 ± 1.02c
Soil pH	8.01 ± 0.01b	8.35 ± 0.02ab	8.03 ± 0.11b	8.44 ± 0.36a	7.94 ± 0.41b
Water content %	24.78 ± 1.01c	25.40 ± 0.74bc	34.92 ± 1.29a	26.54 ± 0.52b	36.42 ± 0.50a

Note: different letters in each line represent significant differences at p < 0.05.

## Conclusions

In this study, a VEE-IPBP system was established through the combination, allocation and regulation of farmland environmental factors, which directly or indirectly affected soil bacterial diversity and community composition. Compared with alterations in soil bacterial Chao, Shannon, and Ace indices, changes in soil dominant bacterial genera such as *Lysobacter* accounted for significantly enhanced suppression of soil-borne diseases and maintained soil health. Moreover, the effect was more significant as the duration increased. Overall, earthworms alter the microbial activity and pH of soil, increase soil aeration by burrowing, alter the form and content of soil as well as nutrients, and directly or indirectly affect soil bacterial diversity and community structure. At the same time, the inclusion of eels also directly or indirectly affects changes in soil bacterial diversity and community structure, but the mechanism requires further study. In addition, the coexistence of water and drought increased the soil moisture content and also affected physical and chemical reactions, the growth of plants through microclimate, and the structure and abundance of soil bacteria.

## Materials and Methods

### General information on the experimental site

The experimental site is located in Sanxing Township (31°41′15″N, 121°54′00″E) on Chongming Island within the municipal area of Shanghai, China. Having risen above the sea surface more than 1,300 years ago, Chongming Island, the largest estuarine alluvial island in the world, has a smooth terrain with an average elevation of 4 m; the soil type on the island is silty saline. Situated in a region with a northern subtropical monsoon climate with prevailing south-easterly winds, Chongming Island is warm and wet with ample sunshine and has four distinct seasons with wet and hot summers and cold and dry winters. Typhoons, storms and droughts are common calamitous weather conditions. Chongming Island has an annual mean precipitation of 1,003.7 mm, with most of the precipitation occurring in April through September, an annual mean temperature of 15.3 °C, an annual mean accumulated temperature of 2,559.60 °C for days with temperature ≥10 °C, a frost-free period of 229 d and an annual sunshine duration of 2,104.0 h.

### Experimental design

**Figure 5:**
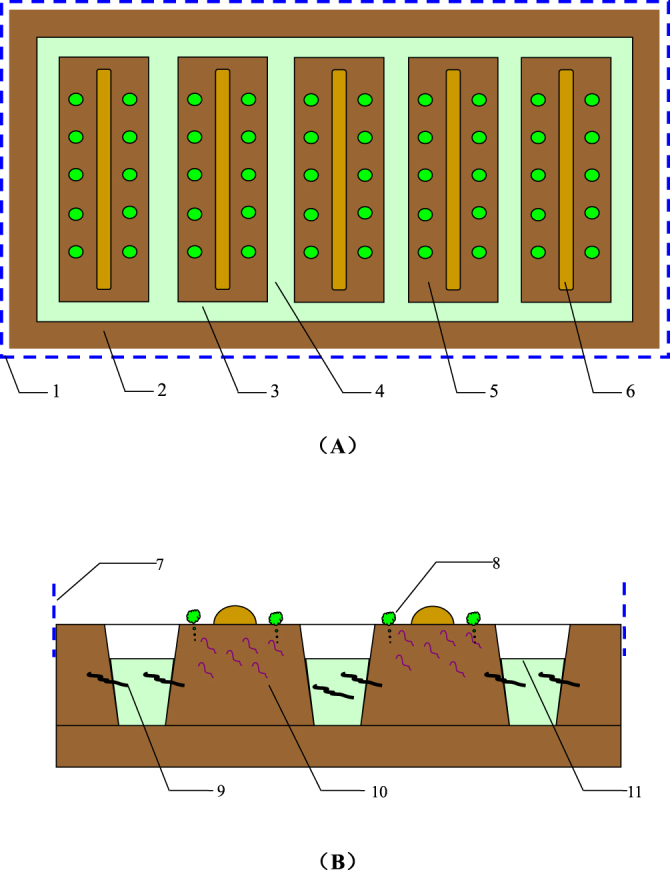
Schematic top (**A**) and sectional (**B**) views of VEE-IPBP (1: blocking net; 2: soil ridge at the vegetable field border; 3: border ditch; 4: inter-furrow ditch; 5: plot surface; 6: soil ridge within the plot; 7: blocking net; 8: vegetable; 9: swamp eel; 10: earthworm; 11: water surface).

The experiment began in June 2010. Two planting systems, namely, a traditional planting systems (TPP) and VEE-IPBP, were used^41^. Each planting systems was implemented in three replicate plots. The plots were randomly arranged in a randomized block. Each VEE-IPBP-treated plot consisted of dry and wet fields in the same space. Dry-farmed vegetables were planted, and earthworms were reared in the soil; swamp eels were reared in ditches (Fig. 5). The specific preparations are described as follows.

#### Vegetable field ditch and plot layout restructuring

In each plot, the vegetable field was surrounded by 100-cm-wide soil ridges at the borders. The ditches were excavated at the borders of and within each plot. Each ditch had an upper opening width of 60 cm, a depth of 60 cm and two sloping sides with an inclination of 60° relative to the ground. Each plot was 600 cm in width. A 40-cm-wide, 20-cm-tall soil ridge was prepared in the centre of each plot. Purse seines were placed along the periphery of the vegetable field to prevent the eels from escaping. In addition, 15% of each *mu* of the field was covered by water.

#### Ditch disinfection and plot fertilization

The newly excavated ditches in the vegetable field were disinfected with quicklime spray. For each hectare of the water surface, 225 kg of quicklime was used. After the quicklime was sprayed, the ditches were filled with water, and the water surface was controlled to remain 15–25 cm below the plot surface. A commercial organic fertilizer consisting of OM (413.4 g∙kg^−1^), nitrogen (N) (17.1 g∙kg^−1^), phosphorous pentoxide (12.4 g∙kg^−1^) and potassium oxide (12.3 g∙kg^−1^) was applied as base fertilizer at a dose of 18 t∙hm^−2^, and a compound fertilizer (15-15-15) (90% as base fertilizer and 10% as a topdressing material) was evenly sprayed at a dose of 375.0 kg∙ha^−2^ onto the vegetable field surface. The same commercial organic fertilizer was applied as base fertilizer at a dose of 15 t∙ha^−2^, and compound fertilizer (15-15-15) (60% as base fertilizer and 40% as topdressing) was evenly sprayed at a dose of 750 kg∙ha^−2^ onto the cauliflower field surface.

#### Earthworm and swamp eel introduction and vegetable planting

Earthworms (each weighed 3 g) were introduced at a density of 120 per m^2^ (a natural density of 60–80 per m^2^ of the surrounding vegetable field). The earthworm species used is *Pheretima guillelmi* (Michaelsen,1895), a native species of Chongming Island. In the course of the experiment, new earthworms were born, and old earthworms were killed or eaten by eels. In late winter and early spring, when the temperature was above 6–10, the number of earthworms in the field was investigated, with more being removed or less added to achieve a density of approximately 120 per square metre. The results of the investigation in 2016 showed a density of 155/m^2^ in treated soil and 66/m^2^ in non-cultivated soil. Eel fry (40 eels per kg) were introduced at a density of 225 kg∙ha^−2^. Each season after vegetable harvesting, the earthworm and swamp eel densities were examined and adjusted to the initial values. Taro and cauliflower were intercropped each year. After April 5th every year, *Monopterus albus* in the furrows of 5 square metres were caught using an eel catch to record density and quality. The taro, cauliflower, earthworm and eel yields and economic benefits are shown in Table 3, with higher values than in the traditional planting model.

**Table 3:** Comparison of yield and benefit of unit farmland under two planting models.

Treatment	Production (kg/667 m^2^)					Economic benefits (rmb/667 m^2^)			
	Broccoli	Taro	Eel	Earthworm	Broccoli	Taro	Eel	Earthworm	Total
TPP10	2356.2	906.2	/	/	2356.2	5437.2	/	/	5437.2
TPP13	2597.3	955.7	/	75.4	2597.3	5734.2	/	1508.0	7242.2
VEE13	2880.8	960.4	9.6	155.5	2880.8	5762.4	1152.0	3110.0	10024.4
TPP16	2708.1	902.8	/	60.8	2708.1	5416.8	/	1216.0	6632.8
VEE16	3003.6	1012.6	13.7	183.7	3003.6	6075.6	1644.0	3674.0	11393.6

Notes: From 2010 to 2016, the average market price of cauliflower was 1 yuan/kg, taro 6 yuan/kg, earthworm 20 yuan/kg, and eel 120 yuan/kg.

### Soil sample collection

Before the experiment began in June 2010, reference soil samples were collected from the vegetable field. These samples are denoted as TPP10-1, TPP10-2 and TPP10-3. Soil samples were also collected from TPP- and VEE-IPBP-treated plots after crop harvesting in November 2013 and November 2016, respectively. The soil samples collected from TPP-treated plots in 2013 and 2016 are denoted as TPP13-1, TPP13-2 and TPP13-3 and TPP16-1, TPP16-2 and TPP16-3 respectively. The soil samples collected from VEE-IPBP-treated plots in 2013 and 2016 are denoted as VEE13-1, VEE13-2 and VEE13-3 and VEE16-1, VEE16-2 and VEE16-3, respectively. All soil samples were collected from the surface layer (0–20 cm) using a stainless steel soil sampler in an “S”-shaped pattern. In each replicate plot, soil samples were collected from 15 different locations and then mixed and placed in a sealed polyethylene bag, which was subsequently stored in a low-temperature preservation box and returned to the laboratory. In the laboratory, impurities (plant and animal residues) were removed, after which the soil samples were sieved through a 20-mesh sieve. Some soil samples were dried and analysed for determination of basic physical and chemical properties; others were subjected to high-throughput sequencing analysis.

### Test items and methods

Determination of physical and chemical properties of soil. The physical and chemical indices for the soil, including OM, TN, TP, TK, AN, AP, AK and moisture contents and pH, were determined according to protocols of *Analytical Methods of Soil Agricultural Chemistry*^42^.

Illumina high-throughput sequencing analysis. Soil samples stored in a box filled with dry ice were transported to Shanghai Biozeron Biotechnology Co., Ltd. for Illumina (rapid-mode, 250-bp, paired-end) high-throughput sequencing analysis. The 515F–907 R primer set (primer sequences: 515 F: GTGCCAGCMGCCGCGG; 907 R: CCGTCAATTCMTTTRAGTTT) was used to amplify the V4-V5 regions of the bacterial 16 S rDNA. Three biological replicates were performed for each soil sample. An ABI GeneAmp® 9700 was used for polymerase chain reaction (PCR) with the following parameters: a) 1 × (5 min at 95 °C); b) 27 × (30 s at 95 °C; 30 s at 55 °C; 45 s at 72 °C); c) 1 × (10 min at 72 °C; 10 °C until halted by user).

### Data processing

#### Analysis of soil physical and chemical properties

One-way analysis of variance was performed using SPSS 13.0 to analyse differences in physical and chemical properties of soil between TPP- and VEE-IPBP-treated plots.

#### OTU clustering analysis

The biological information for OTUs with 97% similarity was statistically analysed based on the USEARCH algorithm^43^.

#### Bacterial diversity analysis

Species richness and diversity indices for bacterial communities were analysed using QIIME^44^.

#### Species composition analysis

Based on datasheets in tax_summary (a document folder), plots were produced using the R language to analyse the distribution of communities in soil samples at phylum and genus levels.

#### Difference analysis

Communities or species leading to a significant difference in sample partitioning were determined using the LDA effective size (LEfSe)^45,46^.

#### Correlation analysis

The canonical correspondence analysis (CCA) algorithm in the vegan package in R software was used to produce plots and analyse relationships among environmental factors, samples and bacterial communities or those between any two environmental factors, samples and bacterial communities^47^.

#### Sequencing data statistics and optimization

Illumina high-throughput sequencing and optimization generated 576,175 sequences from 15 samples obtained from five treatments with different planting systems (TPP or VEE-IPBP) and durations (0, 3 or 6 years) with a total of 216,871,530 bases and an average length of 376.4 bp. The base sequences with a length of 351–400 bp accounted for 99.91% of all sequences (Table 4).

**Table 4:** Length distribution of valid 16s rDNA sequences.

Length (bp)	Sequences	Percent (%)
1–250	2	0.0003
251–300	14	0.0024
301–350	36	0.0063
351–400	575,665	99.9115
401–450	458	0.0795
